# Variation in laboratory testing for patients with long-term conditions: a longitudinal cohort study in UK primary care

**DOI:** 10.3399/BJGPO.2022.0139

**Published:** 2023-01-25

**Authors:** Timothy Jones, Rita Patel, Martha M C Elwenspoek, Jessica C Watson, Ed Mann, Katharine Alsop, Penny F Whiting

**Affiliations:** 1 The National Institute for Health Research Applied Research Collaboration West (NIHR ARC West), University Hospitals Bristol NHS Foundation Trust, Bristol, UK; 2 Population Health Sciences, Bristol Medical School, University of Bristol, Bristol, UK; 3 Musculoskeletal Research Unit, Translational Health Sciences, Bristol Medical School, University of Bristol, Bristol, UK; 4 Tyntesfield Medical Group, Bristol, UK; 5 Nightingale Valley Practice, Bristol, UK; 6 Brisdoc Healthcare Services, Bristol, UK

**Keywords:** general practice, primary health care, hypertension, diabetes mellitus, renal insufficiency, chronic, healthcare disparities

## Abstract

**Background:**

Use of laboratory testing has increased in the UK over the past few decades, with considerable geographical variation.

**Aim:**

To evaluate what laboratory tests are used to monitor people with hypertension, type 2 (T2) diabetes, or chronic kidney disease (CKD) and assess variation in test use in UK primary care.

**Design & setting:**

Longitudinal cohort study of people registered with UK general practices between June 2013 and May 2018 and previously diagnosed with hypertension, T2 diabetes, or CKD.

**Method:**

Clinical Practice Research Datalink (CPRD) primary care data linked to ethnic group and deprivation was used to examine testing rates over time, by GP practice, age, sex, ethnic group, and socioeconomic deprivation, with age–sex standardisation.

**Results:**

Nearly 1 million patients were included, and more than 27 million tests. The most ordered tests were for renal function (1463 per 1000 person-years), liver function (1063 per 1000 person-years), and full blood count (FBC; 996 per 1000 person-years). There was evidence of undertesting (compared with current guidelines) for HbA1c and albumin:creatinine ratio (ACR) or microalbumin, and potential overtesting of lipids, FBC, liver function, and thyroid function. Some GP practices had up to 27 times higher testing rates than others (HbA1c testing among patients with CKD).

**Conclusion:**

Testing rates are no longer increasing, but they are not always within the guidelines for monitoring long-term conditions (LTCs). There was considerable variation by GP practice, indicating uncertainty over the most appropriate testing frequencies for different conditions. Standardising the monitoring of LTCs based on the latest evidence would provide greater consistency of access to monitoring tests.

## How this fits in

Rates of laboratory testing in UK GP practices have been increasing over the past few decades, with considerable geographical variation. This study showed that testing rates to monitor hypertension, T2 diabetes, and CKD have mostly stopped increasing in recent years, but there is still considerable variation by GP practice. Evidence was found of potential undertesting of HbA1c and microalbuminuria levels, and potential overtesting of lipids, FBC, liver function, and thyroid function compared with a review of guidelines. Standardising the monitoring of LTCs based on the latest evidence would provide greater consistency of access to monitoring tests and optimal care for patients.

## Introduction

Rates of laboratory testing have been rising in the UK,^1,2^ with significant geographical variability.^1^ A large proportion of general practice laboratory testing is thought to represent monitoring for LTCs; for example, T2 diabetes, hypertension, and CKD.^3^ By convention, patients with LTCs receive regular laboratory tests to monitor disease progression and response to treatment, and detect complications and side effects of medications. While some of this testing is supported by evidence and guidelines, this is not universally the case;^4^ for example, when testing patterns of 20 primary care practices in North Devon were reviewed, no two practices had the same testing algorithms for monitoring LTCs.^3^ In the context of an increasingly risk-averse society, and with a lack of clear, easy-to-follow guidelines, clinicians may add additional tests for disease monitoring ‘just in case’.^5,6^ There is increasing recognition that some of this testing may be wasteful. The Carter report in 2008 estimated that around 25% of pathology testing overall may be unneccesary^7^ and more recently the Organisation for Economic Cooperation and Development (OECD) estimated that one-fifth of healthcare expenditure is wasted.^8^ In addition, there is increasing strain on the NHS as highlighted by the Care Quality Commission (CQC) report, 2016–2017,^9^ which has been further exacerbated by the COVID-19 pandemic. An Academy of Medical Royal Colleges report has called on doctors to take responsibility for cutting waste, with overuse of laboratory tests being one of three core areas of focus.^10^

As well as being a potential source of waste, overuse of laboratory tests may be a source of harm, potentially causing patient anxiety, unnecessary downstream tests,^11^ referrals, and overdiagnosis. It also has a significant impact on GP workload and costs through reviewing test results and further investigations following abnormal tests.^6^ On the other hand, failure to test may lead to delayed diagnoses, complications, patient harm, and litigation.

Previous studies found an increase in testing in primary care between 2000 and 2015,^1,2^ and wide variation in testing by region of the UK for some tests.^1^ The study objectives were to find out what tests are ordered for patients with common LTCs (hypertension, T2 diabetes, or CKD), describe variation in their use over time by GP practice and patient characteristics, and compare this with current evidence-based guidelines where available.^4^

## Method

This is a longitudinal observational study using prospectively collected routine administrative information about patients registered with UK GP practices from June 2013–May 2018. It is reported according to the REporting of studies Conducted using Observational Routinely Collected health Data (RECORD)^12^ extension to Strengthening the Reporting of Observational Studies in Epidemiology (STROBE) guidelines for observational studies.

### Data sources

Data were from the CPRD GOLD, anonymised health records from approximately 16 million patients at 758 UK general practices over the past 30 years.^13^ CPRD GOLD is representative of the UK general population in terms of age, sex, and ethnic group.^13^ Around 55% of patients were eligible for linkage (by CPRD) to other datasets, and were linked to death certificate information (for accurate dates of death), indices of multiple deprivation, and hospital admissions records (for ethnic group). Linkage is only available for GP practices in England that don’t opt out. Missingness in deprivation and ethnic group was largely owing to linkage ineligibility, and was coded as ‘missing’ without excluding people. Where death certificate information was available, the date of death from the certificate was used, otherwise the CPRD-derived date of death was used. The CPRD pregnancy register was used to determine if people were pregnant during the study period.

### Identifying people with long-term conditions

**Figure 1.**
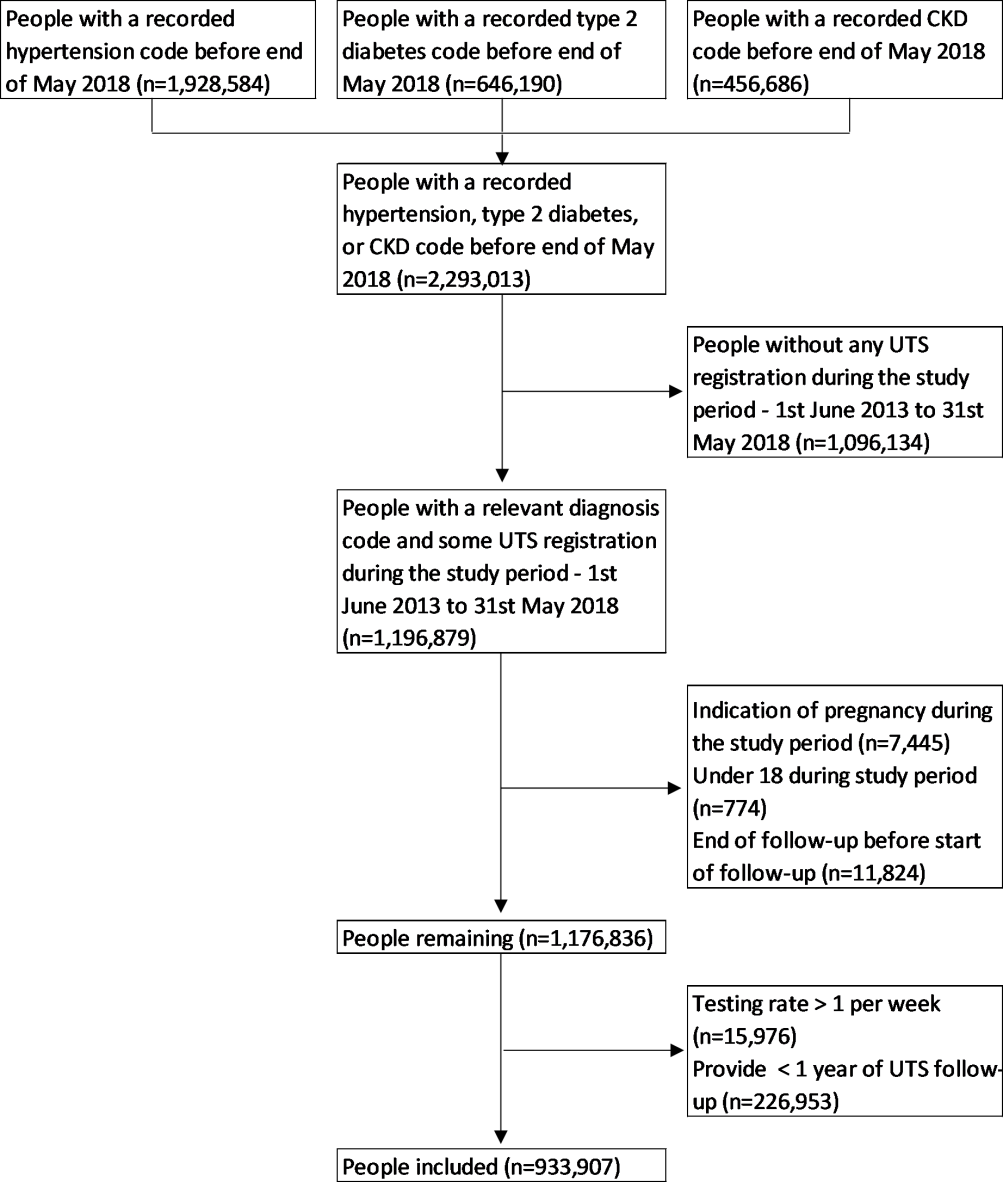
Inclusion and exclusion of people to the cohort with long-term conditions. CKD = chronic kidney disease. UTS = up to standard (GP practice data quality).

The sample (*n* = 1 196 879) comprised people who had active registration at a contributing GP practice during the study period and identified with a code indicating any of three common LTCs in their GP record before 31 May 2018: hypertension (Supplementary Table S1), T2 diabetes (Supplementary Table S2), or CKD (Supplementary Table S3). Exclusions included people providing <1 year of follow-up (*n* = 226 953, 19% of the cohort) to ascertain robust individual testing rates, leaving 933 907 people for analysis (see Figure 1). Demographics for excluded people were like the included cohort (Supplementary Table S4) with less missing information, which was mostly owing to a smaller proportion of people from Scotland, Wales, and Northern Ireland, who were ineligible for linkage and tended to have longer follow-up at the same GP practice.

**Figure 2.**
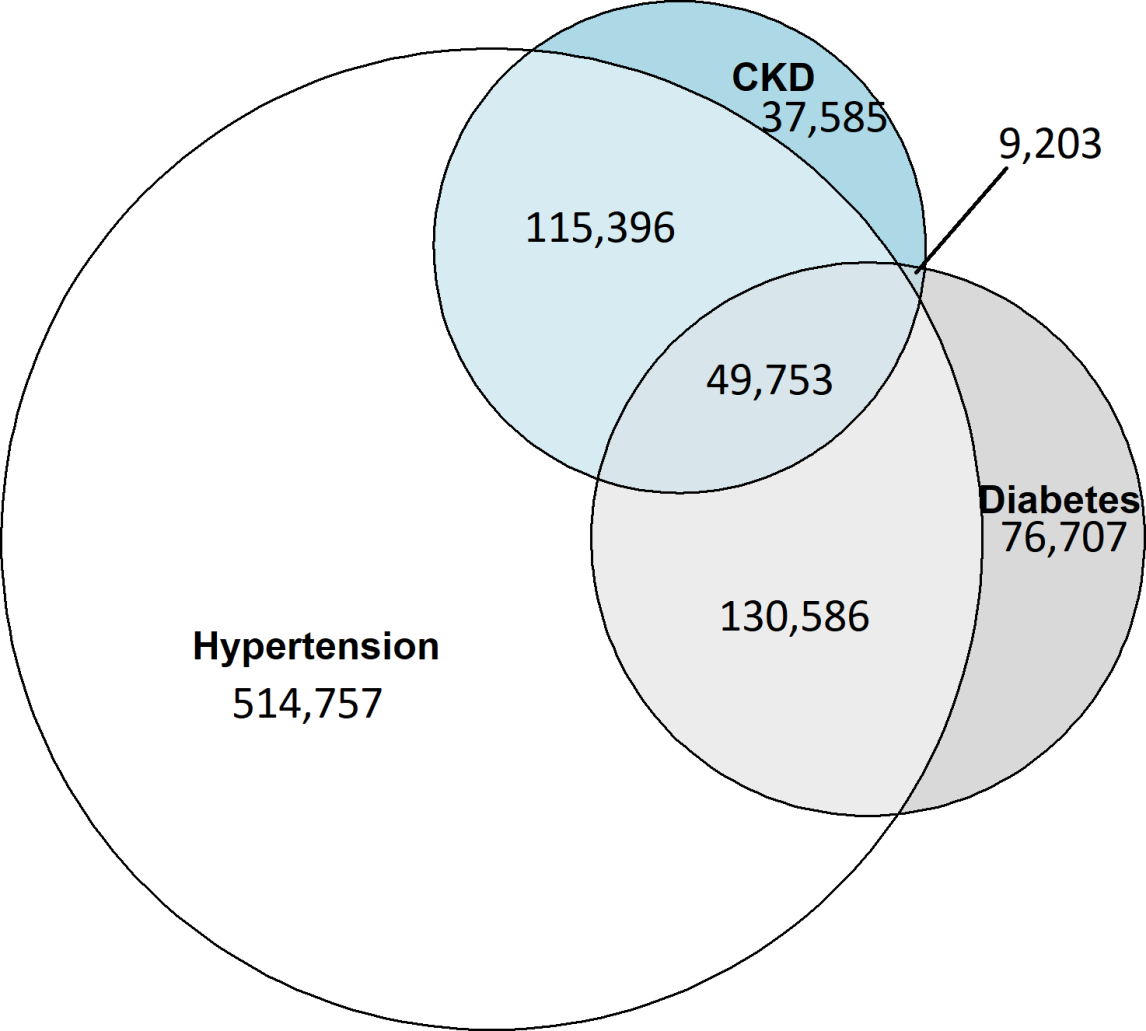
Venn diagram for numbers with each of the three long-term conditions and the overlap between them (933 907 individuals). CKD = chronic kidney disease

**Figure 3.**
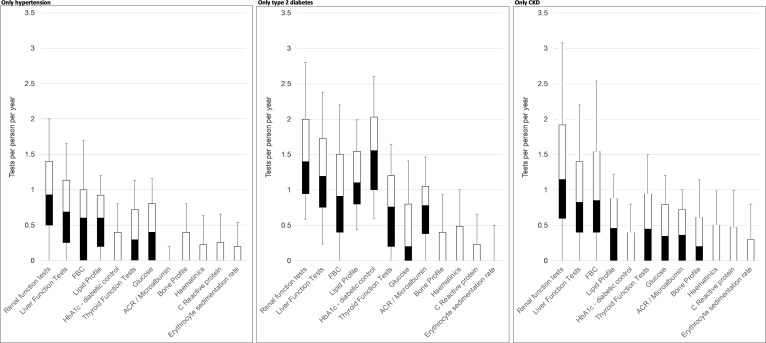
Tests ordered per person per year for people with only hypertension (left), only type 2 diabetes (middle), or only chronic kidney disease (right). Note: Box plot shows median, interquartile range (25th and 75th percentiles), and whiskers are 10^th^ and 90^th^ percentiles.

### Testing rates

Crude testing rates were calculated by dividing the number of tests ordered by the person-years of follow-up. Age–sex standardised testing rates were estimated using direct standardisation^14^ (see Supplementary Box S1 for detailed methods). Testing rates were explored by sex, age group (0–49 years, 50–59 years, 60–69 years, 70–79 years, 80–89 years, ≥90 years), practice, region, year of testing (2013/14 to 2017/18), deprivation quintile, ethnic group (White, Black, Asian, other), time since diagnosis (<1 year and ≥ 1 year), and number of LTCs (1–3). To explore variation in standardised testing rates across GP practices, all of the GP practices by standardised testing rate were ordered from smallest to largest and the rate at the 90^th^ percentile was divided by the 10^th^ percentile rate. This provided a robust indication of variation while ignoring extreme outliers.

### Comparison with testing guidelines

Some of the authors previously conducted a review of laboratory testing guidelines for monitoring hypertension, T2 diabetes, and CKD within the National Institute for Health and Care Excellence (NICE); Scottish Intercollegiate Guidelines Network; Royal Colleges of Pathologists, Physicians, and General Practitioners; and the Quality Outcomes Framework.^4^ The findings are summarised in Figures 1 3 3 of Elwenspoek *et al*,^4^ which were compared with this study’s testing rates.

Supplementary Tables S6-S13 use colour lightness as a guide to the eye to communicate higher or lower rates.^15,16^ All analysis used Stata (version 16.1).^17^ Code lists and Stata code are available at: https://github.com/jonestim2002/primary_care_testing

## Results

### Patient and practice characteristics

Figure 2 shows a Venn diagram of the cohort: 87% (*n* = 810 492) had hypertension; 67% (*n* = 629 049) had only one of the conditions; 27% (*n* = 255 185) had two conditions; and 5% (*n* = 49 753) had all three. Table 1 describes the population demographics stratified by LTC. Sixty-one per cent of people with only CKD were women, while 40% of people with only T2 diabetes were women. Those with only T2 diabetes were younger on average (median age group: 50–59 years), while for people with only CKD the median age group was 70–79 years. There was a deprivation gradient for people with only hypertension or only CKD (25% least deprived to 13% most deprived for both), but not for T2 diabetes (around 20% in each quintile). In addition, 9% of people with only T2 diabetes were Asian, compared with 1% of people with only CKD.

**Table 1.** Patient demographics by long-term condition (*n* = 933 907)

Cohort	All hypertension	Hypertension only	All diabetes	Diabetes only	All CKD	CKD only
*n* (% of cohort)	810 412 (86.8%)	514 757 (55.1%)	266 169 (28.5%)	76 707 (8.2%)	211 937 (22.7%)	37 585 (4.0%)
Average follow-up, years (SD)	3.39 (1.42)	3.33 (1.42)	3.39 (1.43)	3.11 (1.41)	3.40 (1.42)	3.12 (1.4)
Women (%)	417 137 (51.5%)	263 882 (51.3%)	116 323 (43.7%)	30 423 (39.7%)	125 737 (59.3%)	22 970 (61.1%)
Age, years						
0–49	69 253 (8.5%)	56 515 (11.0%)	30 688 (11.5%)	19 652 (25.6%)	5214 (2.5%)	2305 (6.1%)
50–59	132 564 (16.4%)	100 697 (19.6%)	47 622 (17.9%)	20 006 (26.1%)	11 909 (5.6%)	4131 (11.0%)
60–69	198 668 (24.5%)	138 543 (26.9%)	66 105 (24.8%)	18 593 (24.2%)	30 110 (14.2%)	6929 (18.4%)
70–79	210 480 (26.0%)	126 887 (24.6%)	67 268 (25.3%)	12 465 (16.3%)	61 187 (28.9%)	9966 (26.5%)
80–89	150 878 (18.6%)	71 719 (13.9%)	44 291 (16.6%)	5049 (6.6%)	73 170 (34.5%)	9646 (25.7%)
≥90	48 569 (6.0%)	20 396 (4%)	10 195 (3.8%)	942 (1.2%)	30 347 (14.3%)	4608 (12.3%)
Region						
North East	5423 (0.7%)	3380 (0.7%)	1756 (0.7%)	524 (0.7%)	1439 (0.7%)	238 (0.6%)
North West	79 662 (9.8%)	50 351 (9.8%)	27 423 (10.3%)	7857 (10.2%)	20 998 (9.9%)	3679 (9.8%)
Yorkshire and the Humber	7216 (0.9%)	4511 (0.9%)	2560 (1.0%)	767 (1.0%)	1872 (0.9%)	317 (0.8%)
East Midlands	1353 (0.2%)	873 (0.2%)	504 (0.2%)	157 (0.2%)	267 (0.1%)	25 (0.1%)
West Midlands	70 046 (8.6%)	42 454 (8.2%)	23 134 (8.7%)	6383 (8.3%)	21 165 (10.0%)	3605 (9.6%)
East of England	44 093 (5.4%)	29 540 (5.7%)	12 911 (4.9%)	3466 (4.5%)	9424 (4.4%)	1406 (3.7%)
South West	56 990 (7.0%)	34 583 (6.7%)	18 891 (7.1%)	5250 (6.8%)	18 018 (8.5%)	3629 (9.7%)
South Central	86 190 (10.6%)	57 568 (11.2%)	25 465 (9.6%)	7845 (10.2%)	20 362 (9.6%)	3372 (9.0%)
London	82 620 (10.2%)	53 350 (10.4%)	30 412 (11.4%)	9389 (12.2%)	16 867 (8.0%)	2821 (7.5%)
South East Coast	90 307 (11.1%)	55 868 (10.9%)	28 052 (10.5%)	7886 (10.3%)	27 992 (13.2%)	5640 (15.0%)
Northern Ireland	34 257 (4.2%)	21 322 (4.1%)	11 146 (4.2%)	3361 (4.4%)	10 883 (5.1%)	2418 (6.4%)
Scotland	116 790 (14.4%)	74 728 (14.5%)	38 497 (14.5%)	11 904 (15.5%)	30 185 (14.2%)	5054 (13.4%)
Wales	135,465 (16.7%)	86,229 (16.8%)	45,418 (17.1%)	11,918 (15.5%)	32,465 (15.3%)	5381 (14.3%)
Deprivation quintile						
1 — Least	98 840 (24%)	66 295 (25.4%)	26 489 (19.7%)	7474 (19.3%)	25 006 (23.3%)	4697 (24.7%)
2	91 231 (22.2%)	58 934 (22.6%)	27 388 (20.4%)	7560 (19.5%)	23 815 (22.2%)	4280 (22.5%)
3	88 676 (21.6%)	56 040 (21.5%)	28 559 (21.2%)	7896 (20.4%)	23 384 (21.8%)	4100 (21.6%)
4	74 759 (18.2%)	45 819 (17.5%)	27 634 (20.5%)	8222 (21.2%)	19 801 (18.4%)	3398 (17.9%)
5 — Most	57 819 (14.1%)	34 001 (13.0%)	24 430 (18.2%)	7585 (19.6%)	15 499 (14.4%)	2532 (13.3%)
Missing	399 087 (49.2%)	253 668 (49.3%)	131 669 (49.5%)	37 970 (49.5%)	104 432 (49.3%)	18 578 (49.4%)
Ethnic group						
White	313 676 (92.5%)	192 426 (93.2%)	99 936 (87.1%)	26 297 (84.2%)	93 235 (95%)	16 392 (96.5%)
Black	8371 (2.5%)	5068 (2.5%)	3694 (3.2%)	1039 (3.3%)	1597 (1.6%)	176 (1%)
Asian	10 706 (3.2%)	5007 (2.4%)	7946 (6.9%)	2842 (9.1%)	2164 (2.2%)	244 (1.4%)
Other	6453 (1.9%)	3958 (1.9%)	3102 (2.7%)	1068 (3.4%)	1136 (1.2%)	167 (1%)
Missing	471 206 (58.1%)	308 298 (59.9%)	151 491 (56.9%)	45 461 (59.3%)	113 805 (53.7%)	20 606 (54.8%)

CKD = chronic kidney disease.

### Testing rates

For clarity, the study focused on testing rates for people with only hypertension, only T2 diabetes, or only CKD (Figure 3, Supplementary Table S6); information for people with multiple conditions is presented in the Supplementary Tables (S11–S13). The most ordered tests were for renal function (1463 per 1000 person-years), liver function (1063 per 1000 person-years), and FBC (996 per 1000 person-years).

Figure 3 shows the number of tests ordered per person per year for each type of test and each LTC. There was some evidence for undertesting compared with recommendations and guidelines.^4^ ACR testing is recommended 1–4 times per year for T2 diabetes and CKD, whereas testing rates were observed that were lower than once per year for most people, and testing decreased during the study period (by -24% for hypertension to -69% for CKD, Supplementary Tables S7–S9). HbA1c monitoring is recommended every 2–6 months for people with T2 diabetes; while HbA1c testing was highest among the diabetes cohort, most people were tested less than twice per year. HbA1c tests were ordered less than once per year for people with hypertension; annual testing is only recommended for people at high risk of developing diabetes, so this may be appropriate. Evidence was found that estimated glomerular filtration rate (eGFR; kidney function) testing roughly fit with recommendations of 1–4 times per year, although was less frequent for more than half of people with hypertension, nearly half of people with CKD, and around one-quarter of people with T2 diabetes (Figure 3).

There was some evidence for potential overtesting compared with guidelines. Lipid profile testing isn’t recommended among people with hypertension (except with high risk of diabetes) or CKD. However, these tests were recorded around once every 2 years per person, and roughly annually for people with T2 diabetes, presumably to monitor cardiovascular risk.^18^ Liver function testing is not recommended for any of the study's LTCs, so the observed testing rates (median around once per year for T2 diabetes and CKD) may represent overtesting. Additionally, FBC and thyroid function testing rates appear high as these are not routinely recommended except for annual haemoglobin checks (part of FBC) for people with combined T2 diabetes and stage ≥3 CKD.

Supplementary Table S6 shows age and sex standardised testing rates (per 1000 person years) by patient characteristics for the five most common tests, stratified by LTC. Supplementary Tables S7–S9 show the same information for all 12 included tests. The top two rows of each table (number and % tested) show that not everyone with an LTC is receiving tests. Testing increased with age up to the oldest age group (≥90 years) where there was a slight drop off. There were higher testing rates in Scotland and Northern Ireland compared with other regions for most tests. Testing rates were stable over the study period (decreasing overall by 2%), although there was an increase in the use of HbA1c testing in the non-diabetic cohorts (31% for hypertension and 23% for CKD), an increase in haematinics testing (between 36% and 44% increase), and a decrease in the use of blood glucose (between -36% and -38%) and ACR tests (between -30% to -52%). Testing appeared higher among Asian people and lower among Black people compared with White people; and higher for people with more LTCs (that is, sicker people).

### Variation in testing rates

There was considerable variation in testing rates between different people (Figure 2). The percentage of people being tested varied from 13% of the hypertension cohort for ACR or microalbumin testing, to 96% of the diabetes cohort having renal function or HbA1c tests (Supplementary Tables S6–S9). Higher testing practices had up to 27 times higher rates than lower testing practices (that is, HbA1c testing in the CKD cohort; Supplementary Table S6), and even more extreme for erythrocyte sedimentation rate (ESR) tests (Supplementary Tables S7–S9), which were hardly recorded at some practices (<1 test per 1000 person-years).

## Discussion

### Summary

The most common tests for hypertension, T2 diabetes, and CKD were renal function, liver function, FBC, lipid profile, and HbA1c. There was evidence of undertesting of HbA1c and ACR or microalbumin, and potential overtesting of lipids, FBC, liver function, and thyroid function. Some practices had 27 times higher testing rates than others. Overall, testing rates were relatively stable between 2013–2014 and 2017–2018, but increased substantially for some tests (for example, HbA1c among non-diabetic cohorts), and decreased for others (for example, blood glucose and ACR or microalbumin tests). Testing increased with age and comorbidity, and appeared higher among Asian people and lower among Black people compared with White people.

### Strengths and limitations

The size and geographical spread of the cohort should make results generalisable to primary care populations in the UK. Primary care testing was investigated for three common LTCs, which should have relevance to many people. The results were unaffected by recent changes (for example, equipment shortages) owing to the COVID-19 pandemic, as the study period ended before the pandemic began, although this means it reflects older testing rates.

CPRD does not record the reason for ordering a test. Tests used primarily for screening and diagnosis were excluded, but some of the observed ‘overtesting’ of thyroid function, liver function, and FBC may reflect appropriate diagnostic testing of symptoms, rather than monitoring. Given that liver function tests were the second most frequent this seems unlikely to account for all testing observed. There is some variation between laboratories in grouping tests, which may account for some of the observed variation. There may be differences in practice test recording; the study sampled ‘acceptable’ patient records at ‘up-to-standard’ practices to minimise reporting bias. CPRD records some tests initiated in secondary care; it was attempted to exclude tests that were unlikely to be requested by GPs. Testing rates were standardised on age and sex, but other factors may contribute to variation in rates (for example, disease severity). The study has focused on three LTCs, which excludes others such as non-alcoholic fatty liver disease. People with LTCs were identified based on recorded diagnostic codes, which may underestimate the full cohort; however, it is assumed most people with LTCs would have the condition recorded in their medical record. The examination of the impact of comorbidity is limited to the three conditions that were the focus of the study.

There was a lot of missingness in deprivation and ethnic group, which was largely owing to ineligibility of people for linkage. This is a largely descriptive study, and people were included with missing deprivation or ethnic group labelled as ‘missing’ for completeness. As testing rates were not adjusted for variables other than age and sex, there could still be confounding of rates by other factors.

### Comparison with existing literature

Busby *et al* ^1^ found that general practice laboratory test use increased by 24% between 2005 and 2009, while O’Sullivan *et al* ^2^ found an 8.5% increase each year on average between 2000–2001 and 2015–2016; this was slowing over time to 2.6% increase between 2008–2009 and 2015–2016. The results indicate further slowing to near zero growth between 2013–2014 and 2017–2018, with a large increase (more than 100%) of HbA1c testing among hypertension and CKD cohorts, and substantial decreases in blood glucose and ACR or microalbumin testing. The likely intention of increased HbA1c testing is to diagnose and treat diabetes early, which can prevent disease progression and complications.^19^ Reduced blood glucose testing may represent a shift to HbA1c testing for diabetes, and reductions in ACR or microalbumin may be partly owing to their removal from the Quality Outcomes Framework (QOF; removal of indicator CKD004 from 2015/16 onwards),^20^ although testing is lower than NICE recommendations.^4^ Considerable variation was found for certain tests (for example, HbA1c in non-diabetic cohorts), particularly at practice level. The authors of the present study agree with Busby *et al* ^1^ that this may reflect uncertainty about indications for laboratory tests owing to limited clear, evidence-based guidance.^4^

Liver function tests were the second most common in the dataset, despite not being recommended for monitoring any of the LTCs. This may reflect monitoring following prescription of common medications for people with these conditions (for example, statins); liver function tests are recommended at 3 months and 12 months after beginning statin treatment.^21^ The same may be true for lipid profile testing; some evidence suggests natural variation in cholesterol levels makes regular testing less informative,^22,23^ but regular cholesterol checks were encouraged by NICE^18^ and QOF (DM004)^20^ for people with diabetes throughout the study period. The recent NHS Evidence Based Interventions programme recommended reducing lipid and liver function testing following initiation of lipid-lowering therapies.^24^ Thyroid disease monitoring is recommended for people with T1 diabetes; perhaps some observed thyroid testing represents extension of this advice to T2 diabetes.

Health regulation is devolved and testing guidance can be different in England, Wales, Scotland, and Northern Ireland, which could account for higher testing rates in Scotland and Northern Ireland. Higher testing rates among Asian people may relate to the prevalence of LTCs (for example, cardiovascular disease) and multimorbidity in this community.^25^ It is unclear why there should be slightly lower testing rates among Black people; a concern is that this could reflect different access to testing, but more detailed analysis is required to rule out other potential factors.

### Implications for research and practice

Testing in primary care is not increasing as rapidly as it was over the past couple of decades, and this may reflect increasing awareness about the appropriateness of testing. However, there is still variation in testing rates after adjustment for age and sex, particularly at the level of GP practices. Some practices had more than 27 times higher testing rates than others for particular tests (for example, HbA1c in CKD cohort). Some tests were ordered less often than recommended by current evidence-based guidelines (for example, HbA1c, ACR or microalbumin), while others were ordered more often than recommended (for example, liver function tests, FBC, thyroid function) for people with hypertension, T2 diabetes, or CKD. This reflects a lack of clarity or clear communication of the latest evidence-based guidelines for monitoring LTCs. More evidence is needed in terms of patient outcomes and costs to determine the optimum testing levels to maximise population health. The acceptability of testing frequencies to patients and health professionals should be considered. The best way to communicate these recommendations also needs to be ascertained. Standardising the monitoring of LTCs based on the latest evidence would provide greater consistency in access to monitoring tests.

## Data Sharing

This study is based on data from the Clinical Practice Research Datalink (CPRD) obtained under licence from the MHRA and NIHR. The data are provided by patients and collected by the NHS as part of their care and support. CPRD data can be accessed via the MHRA: https://www.cprd.com/

## Data

This study is based on data from the Clinical Practice Research Datalink (CPRD) obtained under licence from the MHRA and NIHR. The data are provided by patients and collected by the NHS as part of their care and support. CPRD data can be accessed via the MHRA: https://www.cprd.com/.
